# Changes in Physical and Oral Function after a Long-Term Care Prevention Program in Community-Dwelling Japanese Older Adults: A 12-Month Follow-Up Study

**DOI:** 10.3390/healthcare9060719

**Published:** 2021-06-11

**Authors:** Yoshimi Iwao-Kawamura, Hideo Shigeishi, Shino Uchida, Shirou Kawano, Tomoko Maehara, Masaru Sugiyama, Kouji Ohta

**Affiliations:** 1 Department of Public Oral Health, Program of Oral Health Sciences, Graduate School of Biomedical and Health Sciences, Hiroshima University, Hiroshima 734-8553, Japan; d204098@hiroshima-u.ac.jp (Y.I.-K.); tmaehara@hiroshima-u.ac.jp (T.M.); masaru@hiroshima-u.ac.jp (M.S.); otkouji@hiroshima-u.ac.jp (K.O.); 2 Bungotakada City Council of Social Welfare, Bungotakada 872-1107, Japan; info@buntaka-syakyo.jp (S.U.); shirou.k.0316@icloud.com (S.K.)

**Keywords:** older adults, physical function, oral function, long-term care prevention programs

## Abstract

Background: The aim of this prospective cohort study was to clarify changes in physical and oral function in older adults after completing a 3-month health program combining physical and oral exercise, oral health instruction, and nutritional guidance. Methods: Subjects were 34 women aged at least 70 years (mean age 79.2 years) in Bungotakada City, Oita Prefecture, Japan. Physical and oral function was investigated on the first day (baseline), at the end of the program, and 6 and 12 months after completing the health program. Physical function was measured using handgrip strength test, timed up and go (TUG) test and one-leg standing time test. Oral diadochokinesis test and repetitive saliva swallowing test (RSST) were employed to assess oral function. Results: TUG scores were significantly lower at 6 and 12 months than at baseline in participants aged ≥70 and <80 years. The repetition rate of the monosyllables */pa/*, */ta/*, and */ka/* was improved at the end of program in participants aged ≥70 and <80 years and increased to more than 6 times/second at 12 months. Conclusions: Our 3-month health program maintained improvements in oral and physical function in older women 1 year after completing the program.

## 1. Introduction

A community-based integrated care system model has been introduced by the Japanese Ministry of Health, Labour and Welfare to ensure that lifestyle assistance, healthcare services, and nursing care are provided in accordance with older adults’ needs [1]. This model supports older adults to remain healthy and live independently in the community, and to continue living in their homes even when they need to receive long-term care [2,3]. Community-based health programs play an important role in facilitating healthy living in older adults. Previous studies have reported that participation in community-based health programs is an important factor in improving oral and physical function in community-dwelling older adults [4,5,6,7].

Since 2015, the local government of Bungotakada City, Oita Prefecture, Japan, has been providing a combined health program, including oral health instruction, physical and oral exercise, and nutritional guidance to older adults living in local communities to promote healthy living [7]. We have previously reported that handgrip strength, walking ability, and oral function (i.e., repetition of monosyllables) were improved in community-dwelling older adults at the completion of this program compared with the baseline [7]. Our results suggest that regular participation in this health program may contribute to improvement in both oral and physical function in community-dwelling older people. Previous research demonstrated that participation in health programs suppressed the deterioration of oral and physical function in community-dwelling older Japanese people [4,6,8,9]. It is assumed that oral and physical function may be maintained or decline after completing participation in a health program in accordance with the participant’s age group. However, it remains unknown how oral and physical function changes after completing a health program (i.e., 6 and 12 months after completing the program) without participation in any further health programs. Furthermore, it remains to be elucidated when the next program should be started after completing a program to maintain oral and physical function. Therefore, we carried out a preliminary prospective cohort study to clarify the changes in physical and oral function in older adults at 6 and 12 months after completion of the program.

## 2. Materials and Methods

### 2.1. Participants

The local government sent letters inviting older adults aged ≥65 years living in local communities to participate in a 3-month health program. A total of 68 older people participated in this health program from May 2019 to February 2021. The study design was approved by the Ethical Committee of Hiroshima University (no. E-1218). All participants signed an informed consent agreement. To exclude older people with a decline in activities of daily living (ADL), older people certified as requiring help or long-term support (n = 10) were excluded. Fifteen participants (2 males and 13 females; mean age, 79.6 years; age range, 68–87 years) dropped out for personal reasons unrelated to the health program (i.e., hospitalization (*n* = 1), leaving the region (*n* = 2), poor health (*n* = 6), or other personal reasons (*n* = 6)) during the 3-month participation period. Men (*n* = 7) were excluded from the analysis because of the low number of male participants. Additionally, participants aged ≥90 years (*n* = 2) were excluded because of the low number of people in this age group. This study therefore included 34 older women (mean age 79.2 years) for analysis. Three participants dropped out 12 months after completing the health program (one in her 70s and two in their 80s; drop-out rate: 8.8%). None of the participants had taken part in any other health programs.

### 2.2. Sample Size Calculations

The sample size required for the Wilcoxon signed-rank tests was calculated using G*Power 3.1.9.4 software (Heinrich Heine Universität, Düsseldorf, Germany) with a statistical power of 80%, a significance level of 5%, and an effect size of 0.5; the sample size was calculated to be 35 participants. Thus, the sample size was considered to be large enough to perform Wilcoxon signed-rank tests. Additionally, a post hoc power analysis was performed using G*Power with a significance level of 5% and an effect size of 0.5. The sample size achieved a statistical power of 0.79.

### 2.3. Physical and Oral Exercise, Oral Health, and Nutritional Guidance Program

A 3-month health program with a weekly intervention in community-dwelling older adults was implemented in this study. The details of the health program, including physical exercise, oral health, and nutritional guidance, were described in our previous study [7]. On the first day of the program (baseline), physical and oral function was examined in each participant, and they were asked to complete a questionnaire about their health condition. The physical exercise program included a stretching exercise, a knee extension and raising exercise, a tiptoe exercise, and a standing-on-one-leg exercise [10]. The oral exercise program included a tongue and lip exercise, salivary gland massage, and tooth brushing instruction [10]. Nutritional guidance included education on cooking techniques, healthy food, and nutrition to prevent malnutrition and dementia [10].

### 2.4. Physical Function Test

Physical function was measured using a handgrip strength test [11], TUG test [12], and a one-leg standing time with eyes open test [9]. The one-leg standing time test was used to measure participants’ ability to stand on one leg with their eyes open for up to 60 s. The TUG test was employed to assess mobility, falling risk, and walking ability.

### 2.5. Oral Function Test

An oral diadochokinesis test was used to evaluate oral motor function [13]. Participants were instructed to pronounce rapid repetitions of the single syllables */pa/*, */ta/*, and */ka/* as quickly as possible. Repetition of the syllable */pa/* reflects lip pressure [14]. Repetition of the monosyllables */ta/* and */ka/* is related to tongue pressure and tongue motor function, respectively [15,16]. The repeated monosyllabic sounds were automatically measured with a counting device (Kenko-kun; Takei Scientific Instruments, Niigata, Japan). Reduced tongue–lip motor function is diagnosed when the number of any counts of */pa/*, */ta/*, or */ka/* produced per second is less than six [17].

The RSST is a screening test for swallowing disorders. The participants were asked to swallow saliva as many times as possible over a 30 s period. Elevation of the larynx during swallowing was counted by the examiner. Fewer than three swallows within 30 s was considered to be a swallowing disorder [18].

### 2.6. Data Collection for Oral Function

The Kihon checklist (KCL), developed by the Japanese Ministry of Health, Labour and Welfare, was employed to evaluate oral frailty. Answers to three questions about difficulty in chewing tough foods, choking or coughing when drinking tea or soup, and feelings of dry mouth and thirst were used to assess oral function [19]. In line with a previous study, participants reporting problems in two or more of these areas were considered to have a decline in oral function [20]. The risk of dysphagia was assessed using the Dysphagia Risk Assessment for the Community-dwelling Elderly (DRACE) [21]. DRACE includes 12 evaluation items; answer options were often (score = 2), sometimes (1), and never (0). A DRACE score of ≥5 was assessed as a high risk of dysphagia; a DRACE score of <3 indicated a low risk of dysphagia [21].

### 2.7. Bacterial Count

The number of oral bacteria was automatically counted using a bacterial counter (Panasonic Healthcare Co. Ltd., Tokyo, Japan) [22]. Oral samples were obtained from the tongue dorsum using a cotton swab in accordance with the manufacturer’s instructions.

### 2.8. Measurement of Oral Moisture Level

Oral moisture levels were assessed using an oral moisture checking device (Moisture Checker Mucus^®^, Scalar, Tokyo, Japan) [23]. Three independent measurements were employed to calculate the mean values of the oral moisture level. A value of ≤27.9 was considered to be oral dryness; a value of ≥29.6 was considered a normal level of wetness [24].

### 2.9. Statistical Analysis

The Mann–Whitney U test was used to evaluate significant differences in clinical factors and physical and oral parameters between the groups (participants aged in their 70s and 80s). Fisher’s exact test was employed to assess significant differences in clinical factors. The Friedman test or Cochran’s Q test were used for multiple comparisons to assess the differences in physical and oral parameters before and after the intervention. The Wilcoxon signed-rank test with Bonferroni correction was used to assess the differences in physical and oral parameters between baseline, the end of program, and 6 months and 12 months after completion of the program. SPSS software, version 24.0 (IBM Corp., Armonk, NY, USA), was employed for statistical analysis. *p*-values of less than 0.05 were regarded as statistically significant.

## 3. Results

### 3.1. Clinical Factors of the Study Population

Table 1 summarizes the participants’ clinical characteristics. To consider the effect of age on physical and oral function, participants were divided into two groups (people aged ≥70 and <80 years and those aged ≥80 and <90 years). A significant difference was found in age between the groups. There were no significant differences in body mass index (BMI), past medical history, denture use, number of remaining teeth, oral bacteria number, or oral moisture level between the groups.

Fisher’s exact test was used to evaluate significant differences in clinical factors between the groups. The Mann–Whitney U test was used to evaluate significant differences in clinical factors such as age, BMI, number of remaining teeth, oral bacterial numbers, and oral moisture level. *p* < 0.05 was considered statistically significant.

### 3.2. Changes in Physical Function in Older Participants

For physical function at baseline, there were significant differences in handgrip strength, TUG scores and one-leg standing time with eyes open between the groups (*p* = 0.043, *p* = 0.006, *p* = 0.001, Mann–Whitney U test). 

Table 2 and Table 3 shows the changes in physical function at baseline, the end of program, and at 6 and 12 months after completion of the program. There was no significant difference in handgrip strength in participants aged ≥70 and <80 years and ≥80 and <90 years (Table 2 and Table 3). A significant difference was found in TUG scores in participants aged ≥70 and <80 years (*p* = 0.002, Friedman test) (Table 2). TUG scores were lower at the end of the program and were significantly lower than baseline at 6 and 12 months in participants aged ≥70 and <80 years (*p* = 0.005 and *p* = 0.03, respectively, Wilcoxon signed-rank test with Bonferroni correction) (Table 2). However, TUG scores were higher at 12 months than at 6 months in this group. There were no significant differences in TUG scores in participants aged ≥80 and <90 years (Table 3). One-leg standing time with eyes open was longer at the end of the program than at baseline in participants aged ≥70 and <80 years, but this increase was not significant. Importantly, one-leg standing time was shorter at 12 months than at 6 months in participants aged ≥70 and <80 years (Table 2). There was no significant difference in one-leg standing time with eyes open in participants aged ≥80 and <90 years (Table 3).

### 3.3. Changes in Oral Function in Older Participants

At baseline, there was a significant difference in the repetition rate of the monosyllable */ta/* between the groups (*p* = 0.017, Mann–Whitney U test). The percentage of participants with a KCL score ≥2 was lower in participants aged ≥70 and <80 years at the end of program, at 6 months, and at 12 months when compared with baseline (Table 4). There was no significant difference in KCL score in participants aged ≥80 and <90 years (Table 5).

There was a significant difference in the repetition rate of the monosyllables */pa/* and */ta/* in participants aged ≥70 and <80 years and those aged ≥80 and <90 years (*p* = 0.005 and *p* = 0.001, *p* = 0.003, and *p* = 0.02, respectively, Friedman test) (Table 4 and Table 5). However, there was no significant difference in the repetition rate of the monosyllable */ka/* in participants aged ≥70 and <80 years and those aged ≥80 and <90 years (Table 4 and Table 5). Importantly, the repetition rate of the monosyllables */pa/*, */ta/*, and */ka/* was higher at the end of program and further increased to more than 6 times/second at 12 months in participants aged ≥70 and <80 years. The repetition rate of the monosyllable */pa/* was significantly higher than baseline in participants aged ≥70 and <80 years at the end of program and at 12 months (*p* = 0.01 and *p* = 0.03, respectively, Wilcoxon signed-rank test with Bonferroni correction) (Table 4). Additionally, the repetition rate of the monosyllable */ta/* was significantly higher than baseline in participants aged ≥70 and <80 years at 6 months (*p* = 0.02, Wilcoxon signed-rank test with Bonferroni correction) (Table 4). The repetition rate of the monosyllable */pa/* was significantly higher than baseline in participants aged ≥80 and <90 years at the end of the program and at 6 months (*p* = 0.02 and *p* = 0.04, respectively, Wilcoxon signed-rank test with Bonferroni correction) (Table 5), and the repetition rate of the monosyllable */ta/* was significantly higher than baseline in participants aged ≥80 and <90 years at 12 months (*p* = 0.045, Wilcoxon signed-rank test with Bonferroni correction) (Table 5). Importantly, the repetition rate of the monosyllables */pa/* and */ta/* was higher at the end of program and further increased to more than 6 times/second at 12 months in participants aged ≥80 and <90 years, but the repetition rate for */ka/* did not increase to more than 6 times/second at any time point (Table 5).

The DRACE score was maintained at less than 3 (i.e., a low risk level of dysphagia) at the end of program and at 6 months and 12 months in participants aged ≥70 and <80 years, but this decrease was not significant (Table 4). In contrast, the DRACE score did not drop below 3 at the end of program or at 12 months in participants aged ≥80 and <90 years (Table 5).

The RSST increased from baseline to nearly three swallows (i.e., a level indicating a low risk of dysphagia) in participants aged ≥70 and <80 years at the end of program and at 6 and 12 months, but this difference was not significant (Table 4). In contrast, RSST increased from baseline to more than three swallows at the end of program and at 6 and 12 months in participants aged ≥80 and <90 years (Table 5). Significant differences were found between baseline and the end of program and at 6 months (*p* = 0.04 and *p* = 0.03, respectively, Wilcoxon signed-rank test with Bonferroni correction) (Table 5).

### 3.4. Changes in Oral Bacterial Numbers and Moisture Levels

At baseline, there were no significant differences in oral bacterial numbers and moisture levels between the groups. There was a significant difference in oral bacterial numbers in participants aged ≥70 and <80 years (*p* < 0.001, Friedman test). Oral bacterial numbers were lower in participants aged ≥70 and <80 years at 6 and 12 months when compared with baseline (Table 6), and a significant difference was found between the end of the program and 12 months (*p* = 0.02, Wilcoxon signed-rank test with Bonferroni correction). Oral bacterial numbers were also lower at the end of the program and at 6 months and 12 months than at baseline in participants aged ≥80 and <90 years, but these differences were not significant (Table 7). There were no significant differences in oral moisture levels in participants aged ≥70 and <80 years and those aged ≥80 and <90 years (Table 6 and Table 7). The oral moisture level was lower than the normal level at each measurement point in both participant groups.

## 4. Discussion

In Japan’s super-aged society, it is vital to prevent the deterioration of physical and oral function in community-dwelling older people to enable them to maintain a healthy lifestyle. It is thought that a multimodal approach, including physical exercise, oral exercise, oral health instruction, dietary advice, and psychosocial health care may be effective in improving the health of older adults [25,26,27]. Therefore, the local government of Bungotakada City has started providing a combined health program that includes oral health instruction, physical and oral exercise, and nutritional guidance.

Our prospective study revealed that mobility and walking ability was significantly improved at 6 months after completing the intervention in participants aged ≥70 and <80 years (i.e., the younger group). Participation in our health program may improve physical functions such as mobility and reduce the risk of falls even after completion of the health program in independent older adults. An individual personalized rehabilitation program over 7 weeks improved TUG scores in non-disabled older people [28]. However, the TUG scores were higher at 12 months than at 6 months in our study, indicating that the improvement in walking ability had begun to decline after 6 months. Lower body muscle strength may decline within 1 year in older people even if it had previously improved. Lower body muscle strength declines faster than upper body muscle strength in older people and is importantly related to poor physical function [29,30]. Therefore, a regular physical exercise program is recommended to maintain walking ability. Additionally, the improvement in one-leg balance had deteriorated at 12 months in participants aged ≥70 and <80 years. Improvements in physical function may start returning to the original level at 6 months after completion of the health program if no additional interventions are provided. Therefore, regular participation in a health program (i.e., at least every 6 months after the intervention) should be considered to maintain the improvements achieved in physical function. In contrast, participants aged ≥80 and <90 years exhibited little or no significant improvement in physical function as measured by the TUG test and the one-leg standing test even after our intervention. Therefore, intervention from a younger age (i.e., <80 years) is important to effectively improve physical function in older people.

The tests for oral function revealed that repetition of the monosyllables */pa/* and */ta/* was at a normal level at 12 months in participants aged ≥80 and <90 years (i.e., the older group), indicating that a decline in lip motor function and tongue pressure can be improved gradually after completion of the program in the older group. Our health program is effective in improving lip motor function and tongue pressure even in adults aged ≥80 years. Repetition of the monosyllable */ka/* reached a normal level at 12 months in participants aged ≥70 and <80 years (i.e., the younger group). However, the monosyllable */ka/* was below normal levels at 12 months in the older group. These results suggest that tongue motor function is more readily improved in the younger group. Therefore, intervention from a younger age (i.e., <80 years) is required to improve tongue motor function in older people. In contrast, a previous study of older Japanese people with a risk of deterioration in oral health found that articulation of the monosyllables */pa/*, */ta/*, and */ka/* improved after participation in a 3-month health program [8]. The average score for the monosyllables */pa/*, */ta/*, and */ka/* was more than 6 times/second (i.e., a normal level) before the intervention [8]. Therefore, the ability to repeat monosyllables before the intervention may be associated with the recovery speed of such ability after intervention.

The KCL scores indicated that the percentage of people with oral frailty was lower in participants aged ≥70 and <80 years after completing the health program, suggesting that the health program may be associated with improvements in chewing and swallowing ability. However, no such reduction in the oral frailty rate was evident in participants aged ≥80 and <90 years after completion of the health program. Individual oral health status (e.g., denture fit and periodontal disease) may also be related to oral function (i.e., mastication ability) in older adults. However, the relationship between oral health status and oral function could not be elucidated in this study. In addition to participation in the health program, proper dental care (i.e., denture treatment and periodontal therapy) should be recommended in accordance with each individual’s oral health status for effective improvement in oral function.

The DRACE scores indicated that participants aged ≥80 and <90 years were at a high risk of dysphagia at baseline. Dysphagia is a serious problem in many community-dwelling older people because many age-related physiological changes increase the risk of dysphagia [31]. A previous study revealed that nearly half of community-dwelling older people were at a risk of dysphagia [6]. Therefore, effective intervention is necessary to improve or maintain swallowing function in older people. Importantly, participants aged ≥70 and <80 years exhibited a low risk of dysphagia after completing the health program. Additionally, RSST scores also dropped almost to a low risk of dysphagia at the end of program and at 6 and 12 months in participants aged ≥70 and <80 years. Furthermore, participants aged ≥80 and <90 years achieved RSST scores indicating a low risk of dysphagia even at 6 and 12 months after completing the program. These results highlight the importance of our health program in improving the swallowing function of older women. The resting position of the hyoid bone tends to be higher in Japanese women than in Japanese men [32]. A higher position may be associated with a lower risk of dysphagia. Therefore, older women may have a lower risk of dysphagia than older men, regardless of the age-related hyoid bone resting position. It is important to consider sex-related differences when evaluating dysphagia risk. Overall, our health program may reduce the risk of dysphagia in older women even 12 months after completing the program.

Oral bacterial numbers were lower at 12 months after completing the intervention than at baseline in participants aged ≥70 and <80 years and those aged ≥80 and <90 years in this study. These results suggest that the oral health intervention may have affected the participants’ oral hygiene status in both the younger and older groups. However, the participants’ plaque accumulation and periodontal health condition were not investigated in this study. A previous study found that the oral candida positive rate and the candida colony score were lower in older Japanese people after participation in a 3-month health program [8]. This result suggests that the oral bacterial count may be reduced in accordance with the improved oral hygiene status immediately after completing the program. However, it remains to be elucidated why the number of oral bacteria was significantly lower at 12 months after completing the intervention in our study. It is assumed that a continuous improvement in oral health resulting from oral hygiene instruction may have caused the oral bacterial count to decrease gradually over a year. Therefore, further investigation will be required to clarify the relationship between the oral bacterial count and oral hygiene status after completion of the intervention.

Previous studies demonstrated that annual regular interventions improved oral and physical function and inhibited deterioration of such functions in older people [33,34]. In our study, improvement in walking ability had declined at 12 months after completing the intervention in participants aged ≥70 and <80 years. Therefore, interventions at least every year may be required to maintain physical functions such as walking ability.

Our results suggest that a 3-month combination program, including physical exercise, oral health instruction, and nutritional guidance, may contribute to the improvement and maintenance of oral and physical function in older women. However, one limitation of this study was that we did not include a control group that was not offered the intervention. To demonstrate the effectiveness of our health program, a comparison of physical and oral function between the intervention group and a control group is required. Further limitations were that the participants were independent older women and that the overall number of participants was small. Future research should investigate the program’s efficacy among a large cohort of older men and women.

## 5. Conclusions

This preliminary prospective study revealed that participation in our 3-month health program improved a marker of physical and oral function at the end of program in independent older women aged ≥70 and <80 years. Improved oral function (i.e., lip–tongue motor function) and physical function (i.e., walking ability) was maintained until 12 months after completing the program. Older women who completed our health program maintained their improved oral and physical function 1 year after the end of the program. For maintenance of improved oral and physical function in older people, regular participation in a health program should be considered.

## Figures and Tables

**Table 1:** Clinical characteristics of participants.

	Participants Aged ≥70 and <80 Years (*n* = 19)	Participants Aged ≥80 and <90 Years (*n* = 15)	*p*-Value
Mean age	75.8 ± 2.7	83.5 ± 2.4	<0.001
BMI (kg/m^2^)	24.0 ± 3.1	23.0 ± 2.6	0.35
Hypertension			
No (17)	12 (70.6%)	5 (29.4%)	0.17
Yes (17)	7 (41.2%)	10 (58.8%)	
Diabetes			
No (28)	17 (60.2%)	11 (39.3%)	0.37
Yes (6)	2 (33.3%)	4 (66.7%)	
Heart disease			
No (31)	19 (61.3%)	12 (38.7%)	0.08
Yes (3)	0 (0.0%)	3 (100%)	
Lipid disorder			
No (27)	15 (55.6%)	12 (44.4%)	1.0
Yes (7)	4 (57.1%)	3 (42.9%)	
Bone-related disease			
No (28)	16 (57.1%)	12 (42.9%)	1.0
Yes (6)	3 (50.0%)	3 (50%)	
Number of remaining teeth	16.2 ± 10.0	10.5 ± 10.4	0.12
Denture use			
Non (7)	6 (85.7%)	1 (14.3%)	0.20
Partial denture user (14)	7 (50.0%)	7 (50.0%)	
Full denture user (13)	6 (46.2%)	7 (53.8%)	
Oral bacteria number (1.0 × 10^6^ [CFU]/mL)	14.6 ± 12.1	20.2 ± 18.2	0.63
Oral moisture level	27.1 ± 3.1	26.7 ± 3.8	0.95

**Table 2:** Changes in physical function in older participants aged ≥70 and <80 years.

	Participants Aged ≥70 and <80 Years (*n* = 19)				
Physical Function Measures	Baseline (*n* = 19)	The End of Program (*n* = 19)	6 Months (*n* = 19)	12 Months (*n* = 18)	^†^*p*-Value
Handgrip strength (kg)	20.2 ± 4.1	20.0 ± 3.7	19.6 ± 3.6	19.6 ± 4.1	0.25
TUG score (second)	8.6 ± 1.3	8.0 ± 1.4	7.7 ± 1.3 ^a^	8.0 ± 1.3 ^b^	0.002
One-leg standing time with eyes open (second)	31.6 ± 18.8	37.6 ± 19.8	37.0 ± 19	34.4 ± 18.8	0.35

^†^ Friedman test. ^a^ Significant difference between baseline and 6 months (Wilcoxon signed-rank test with Bonferroni correction). ^b^ Significant difference between base line and 12 months (Wilcoxon signed-rank test with Bonferroni correction). *p* < 0.05 was considered statistically significant.

**Table 3:** Changes in physical function in older participants aged ≥80 and <90 years.

	Participants Aged ≥80 and <90 Years (*n* = 15)				
Physical Function Measures	Baseline (*n* = 15)	The End of Program (*n* = 15)	6 Months (*n* = 15)	12 Months (*n* = 13)	^†^*p*-Value
Handgrip strength (kg)	16.9 ± 4.3	16.5 ± 5.0	15.5 ± 5.3	15.6 ± 5.9	0.25
TUG score (second)	10.8 ± 2.7	10.4 ± 2.0	10.5 ± 2.5	10.6 ± 2.1	0.90
One-leg standing time with eyes open (second)	11.7 ± 12.4	12.5 ± 11.4	11.0 ± 10.8	8.2 ± 9.1	0.39

^†^ Friedman test. *p* < 0.05 was considered statistically significant.

**Table 4:** Changes in oral function in participants aged ≥70 and <80 years.

	Participants Aged ≥70 and <80 Years (*n* = 19)				
Oral Function Measures	Baseline (*n* = 19)	The End of Program (*n* = 19)	6 Months (*n* = 19)	12 Months (*n* = 18)	^†^*p*-Value
KCL score (oral function)					
0 or 1	12 (63.2%)	13 (68.4%)	17 (89.5%)	14 (77.8%)	0.14
≥2	7 (36.8%)	6 (31.6%)	2 (10.5%)	4 (22.2%)	
Oral diadochokinesis (times/second)					
Pa	6.2 ± 0.7	6.5 ± 0.7 ^a^	6.5 ± 0.6	6.5 ± 0.5 ^c^	0.005
Ta	6.2 ± 0.7	6.3 ± 0.6	6.4 ± 0.7 ^b^	6.3 ± 0.7	0.001
Ka	5.8 ± 0.9	5.9 ± 0.7	5.9 ± 0.8	6.0 ± 0.7	0.20
DRACE score	3.3 ± 2.5	2.9 ± 2.7	2.5 ± 2.1	2.4 ± 2.1	0.23
RSST (times/30 s)	2.0 ± 1.7	2.9 ± 1.3	2.6 ± 1.3	2.8 ± 1.7	0.04

^†^ Cochran’s Q test was used to evaluate significant differences in KCL score. Friedman test was used to evaluate significant differences in oral diadochokinesis, DRACE score, and RSST. ^a^ Significant difference between baseline and the end of the program (Wilcoxon signed-rank test with Bonferroni correction). ^b^ Significant difference between baseline and 6 months (Wilcoxon signed-rank test with Bonferroni correction). ^c^ Significant difference between baseline and 12 months (Wilcoxon signed-rank test with Bonferroni correction). *p* < 0.05 was considered statistically significant.

**Table 5:** Changes in oral function in participants aged ≥80 and <90 years.

	Participants Aged ≥80 and <90 Years (*n* = 15)				
Oral Function Measures	Baseline (*n* = 15)	The End of Program (*n* = 15)	6 Months (*n* = 15)	12 Months (*n* = 13)	^†^*p*-Value
KCL score (oral function)					
0 or 1	10 (66.7%)	8 (53.3%)	10 (66.7%)	9 (69.2%)	0.85
≥2	5 (33.3%)	7 (46.7%)	5 (33.3%)	4 (30.8%)	
Oral diadochokinesis (times/second)					
Pa	5.7 ± 0.9	5.9 ± 0.9 ^a^	6.2 ± 0.7 ^b^	6.2 ± 0.8	0.003
Ta	5.3 ± 1.3	5.9 ± 0.8	5.9 ± 0.7	6.0 ± 1.0 ^c^	0.02
Ka	5.4 ± 0.7	5.5 ± 0.7	5.7 ± 0.7	5.5 ± 0.9	0.1
DRACE score	2.7 ± 2.5	3.1 ± 2.0	2.7 ± 2.3	3.1 ± 2.4	0.39
RSST (times/30 s)	1.9 ± 1.8	3.1 ± 1.8 ^a^	3.2 ± 1.3 ^b^	3.0 ± 1.2	0.007

^†^ Cochran’s Q test was used to evaluate significant differences in KCL score. Friedman test was used to evaluate significant differences in oral diadochokinesis, DRACE score and RSST. ^a^ Significant difference between baseline and the end of the program (Wilcoxon signed-rank test with Bonferroni correction). ^b^ Significant difference between baseline and 6 months (Wilcoxon signed-rank test with Bonferroni correction). ^c^ Significant difference between baseline and 12 months (Wilcoxon signed-rank test with Bonferroni correction). *p* < 0.05 was considered statistically significant.

**Table 6:** Changes in oral bacterial numbers and moisture levels in participants aged ≥70 and <80 years.

	Participants Aged ≥70 and <80 Years (*n* = 19)				
	Baseline (*n* = 19)	The End of Program (*n* = 19)	6 Months (*n* = 19)	12 Months (*n* = 18)	^†^*p*-Value
Oral bacteria number (1.0 × 10^6^ [CFU]/mL)	14.6 ± 12.1	15.5 ± 14.4	14.1 ± 10.6	7.8 ± 10.6 ^a^	<0.001
Oral moisture level	27.1 ± 3.1	27.1 ± 3.0	26.4 ± 2.4	26.6 ± 2.3	0.53

^†^ Friedman test. ^a^ Significant difference between the end of the program and 12 months (Wilcoxon signed-rank test with Bonferroni correction). *p* < 0.05 was considered statistically significant.

**Table 7:** Changes in oral bacterial numbers and moisture levels in participants aged ≥80 and <90 years.

	Participants Aged ≥80 and <90 Years (*n* = 15)				
	Baseline (*n* = 15)	The End of Program (*n* = 15)	6 Month s (*n* = 15)	12 Months (*n* = 13)	^†^*p*-Value
Oral bacteria number (1.0 × 10^6^ [CFU]/mL)	20.2 ± 18.3	14.1 ± 11.5	16.0 ± 10.3	12.4 ± 9.9	0.28
Oral moisture level	26.7 ± 3.8	28.1 ± 2.5	28.7 ± 2.5	27.2 ± 2.0	0.24

^†^ Friedman test. *p* < 0.05 was considered statistically significant.

## Data Availability

All data generated or analyzed in this study are included in this article.
